# The Relationship between Daily Behavior Changes and Vaccine Attitudes at the Early Stage of the COVID-19 Pandemic among Japanese People from Different Demographics: A Retrospective and Exploratory Examination Using a Free-Response Survey

**DOI:** 10.3390/vaccines11010192

**Published:** 2023-01-16

**Authors:** Mariko Kikutani, Mie Matsui, Yuta Takiguchi

**Affiliations:** Institute of Liberal Arts and Science, Kanazawa University, Kanazawa 920-1192, Ishikawa, Japan

**Keywords:** SARS-CoV-2, behavior change, free-response survey, demography, vaccine attitudes

## Abstract

This study investigated how daily behaviors of Japanese people changed during the early stages of the COVID-19 pandemic and whether the change was mediated by demographics. It also examined whether the magnitude of behavior change in a demographic group is related to their attitudes towards the COVID-19 vaccine. 301 Japanese responded to an online survey in February 2021, in which they first wrote some activities they frequently performed before the virus outbreak and then wrote about activities in their current life. The number of gathered answers were 1858 for ‘before’ and 1668 for ‘after’, and they were grouped into 19 behavior categories. Overall, behaviors such as traveling, eating out, and shopping were much less frequently described in the ‘after’ condition; while housework, food delivery, and pandemic prevention were mentioned more. However, the change pattern was significantly influenced by demographics of age, gender, having children or not, and household income. Especially women, younger generations, and people without children showed the greatest extent of behavior change compared with the other demographic cohorts. These groups were reported to be vaccine-hesitant in the literature. This study suggests that individuals with hesitant attitudes towards vaccines are more willing to change their behaviors to control viral transmission.

## 1. Introduction

The spread of COVID-19 started around January 2020, and it has changed the lifstyles of people all over the world since then [1]. The occurrence of behavior change was especially drastic during the early stages of the pandemic, brought about by strict restrictions on movement and inter-individual physical contact [2] and the implementations of various other prevention measures [3]. The current study focused on changes brought about in Japanese individuals’ daily behaviors during the early stages of the pandemic and, although retrospectively, attempted to reveal the relationship between the behavior changes and vaccination attitudes.

In terms of COVID-19-related behavior changes, many studies in recent years have dealt with this topic, especially in the field of mental health. For example, participation in physical exercise has significantly reduced since the virus outbreak, which is strongly associated with mental health deterioration [4,5,6]. On the contrary, time spent on sedentary behaviors and internet use greatly increased, which can enhance mental illness symptoms [5,7]. Another line of research on behavior change focuses on prevention behaviors, such as mask wearing and hand washing. While engaging in prevention behaviors is becoming a new social norm worldwide [8,9,10], the extent of engagement appears to be influenced by age [11], perceived risk of COVID-19 [12], and personality [13].

Instead of investigating the psychological impacts of behavior change, the present research aimed to describe the dynamics of changes experienced by people according to age, gender, family structure and socio-economic background. The study aims to reveal how these demographic features mediate the behavior changes caused by the pandemic. It has been reported that behavior change is influenced by demographics; for example, the pandemic has reduced our opportunities to exercise [6], but females engage in physical exercises generally less than males [14]. Therefore, such demography-based general tendencies must be taken into account when assessing the influence of the pandemic.

Aside from revealing Japanese individuals’ behavior changes at the early stages of the pandemic, this study also aimed to investigate the relationship between these behavior changes and attitudes towards the COVID-19 vaccine. Vaccination is the essential strategy to contain the disease; therefore, investigating people’s attitudes towards vaccination programs has been a popular research topic worldwide in recent years. The Japanese vaccination program has been successful, and the majority of the population has received three doses as of October 2022 [15]. However, many people were hesitant towards vaccines during the early stages of the pandemic [16], and these vaccination attitudes differed according to demographics, such as gender, marital status, and income [17]. The present research retrospectively investigated whether behavior changes and vaccine attitudes during the early stages of the pandemic were related. It can be assumed that a demographic group who were reluctant to show behavior changes might have been more willing to receive the vaccine because it helped them persist in their pre-pandemic lifestyle. On the contrary, a group of people who had hesitant attitudes toward the vaccine might choose to change their behaviors dramatically to compensate for not receiving a vaccine. Research conducted in the US has reported that specific types of COVID-19 prevention behaviors, such as mask usage and social distancing, which are publicly performed and become a part of an individual’s COVID-19 social identity, are strongly associated with vaccine intention. In their study, individuals who are reluctant to perform prevention behaviors tended to have negative intentions toward vaccination [18]. The present research aimed to cover a wide range of behavior changes, not limited to specific prevention behaviors such as mask wearing and hand washing.

In order to reveal changes that occurred for a wide variety of activities, this research used a free-response method, in which participants freely wrote down the activities they frequently performed in everyday life. This method is relatively unique among the research on COVID-19-related behavior changes. It is common for researchers to target a limited number of behaviors as it is difficult to investigate many behaviors at once. Thus, the most frequently used method to assess behavior change in pandemic-related research is to present participants with multiple-choice questions about preselected behaviors. The questions are often about the frequency or duration of specific activities, and participants select answers corresponding to the periods before and during the pandemic [4,5,6,19,20]. Such a method is insufficient to reveal the dynamic changes people have experienced; therefore, the present research chose to use a free-response method.

In the study, which took place in February 2021 (after the pandemic outbreak), approximately 300 Japanese adults reported activities they often performed before the pandemic and at the time of the study. The answers were categorized based on activity type, and the number of answers for the before- and after-outbreak conditions were compared. This procedure does not limit the target behaviors to those in the mind of the researchers. Although this method does not precisely record the frequency of particular actions being performed, the responses should reflect the subjective significance of the behaviors to the participant, as well as the frequency of occurrence. Consequently, the results provide a broader picture of behavioral changes experienced by people from various demographics.

The obtained results were then contrasted with the vaccine attitude using the literature which reported the attitudes among Japanese at roughly the same time as the data collection of the present research (see details in Section 2.4). It was consistently found that demographic characteristics associated with hesitant attitudes at that time were: being women, being young, having low income or assets, and having no children [17,21,22,23,24,25]. In terms of behavior changes, those who were hesitant about the vaccine were likely to choose behavioral strategies to prevent viral spread. Thus, the aforementioned demographic groups might have experienced more behavioral change compared with people belonging to other demographic groups.

Recently, more research has been devoted to predicting the trajectory of epidemic progression using mathematical models [26,27,28,29,30]. Those models often contain various parameters, such as administration of lockdowns in the community, health and socio-economic characteristics of the population, and vaccine implementation. The research has highlighted that socio-demographic factors crucially affect the outcomes of the epidemic because individuals in different social groups behave differently, varying their potential to infect others. Importantly, some research has revealed that the influence of demographics can even be more crucial after the onset of vaccination [30]. Not all the vaccine recipients will be immune and there will be new virus strains that reduce the effectiveness of the vaccines [29]. Therefore, issues such as which demographics are vaccinated first, how many people are vaccinated at a time, how effective vaccines are, etc., affect epidemic progression. In this context, a factor called non-pharmaceutical interventions, meaning various prevention measures (e.g., mask wearing and social distancing) taken by individuals, are regarded as a major determinant of epidemic outcomes alongside vaccines [31,32]. However, the extent of participation in non-pharmaceutical interventions differs immensely between individuals; thus, it is difficult to consider this factor in mathematical models [30]. The present research focuses on individuals’ behavior changes, which reflect their motivation regarding non-pharmaceutical interventions to some extent. Finding relationships between the behavior change pattern and vaccine attitudes among certain demographics can be helpful for the modeling research to parametrize individuals’ epidemic-relevant behaviors.

In summary, the present study has three purposes: (1) investigating Japanese people’s daily behavior change before and after the pandemic onset, (2) examining the impact of demographics on behavior change, and (3) comparing the extent of behavior change and vaccine attitudes by demographic groups.

## 2. Materials and Methods

### 2.1. Participants

Three hundred and twenty-one Japanese adults took part in the survey. They were 160 men and 161 women with a mean age of 47.41 (SD = 19.17). They were registered members of a survey company Macromill (https://group.macromill.com/, accessed on 15 December 2020). The researchers asked the company to contact their members whose age was between 18 to 79 for survey participation. The company distributed the survey in an online format to their randomly chosen members using emails and online advertisement. The completed survey data was sent to the company. They kept on distributing the survey until a target number of data was obtained, which was approximately 300 (see below). The respondents were reimbursed by the company.

The target number of participants was determined based on the planned statistical analysis, which is a multiple regression with five predictor variables and one control variable (the analysis reported in Section 3.3). For this analysis, 92 participants are required for a medium effect size (*f*^2^ = 0.015, *α* = 0.05, 1 − *β* = 0.08). In order to collect sufficient data from people with different ages and to secure some variations in demographic combinations, the target sample size was increased to 300.

### 2.2. Procedure

The survey was conducted online. Participants first reported their demographic information. The questions asked about their gender, age, marital status, whether they have children or not, annual household income, individual annual income, region of residence (prefecture), and occupation. Next, the participants were asked to recall their life before the pandemic and write down at least five (up to ten) activities they frequently performed. Once this was done, they were asked to do the same about their current life. The true purpose of the survey (comparing behaviors before and after the outbreak) was concealed from the participants. Knowing the true purpose may have limited their responses to pandemic-related behaviors. They were informed that the purpose of this study was to identify frequently performed behaviors in a society. The survey was produced online, and the survey company distributed it to the participants.

### 2.3. Dependent Variables and Statistical Analyses

#### 2.3.1. Appearance-Rate Observations

The present research reported various analyses performed on different dependent variables. Table 1 summarizes the variables and analyses reported in each subsection of Results.

The first part of the results section concerned the total number of described behaviors in the ‘before’ and ‘after’ conditions. All the answers by the participants were gathered and they were classified into 19 behavior categories. The number of answers in each category was counted for the ‘before’ and ‘after’ conditions separately. Next, the number of items in each category was divided by the number of participants (*N* = 321). The value was named as an appearance rate. The appearance rates basically indicated the proportion of participants who reported the activities that belong to each of the 19 behavior categories.

The extent of behavior change was examined by comparing the appearance rates for the before and ‘after’ conditions. The overall change from all the data was examined first, and then the change was calculated based on participants’ demographics (age, gender, marital status, having children or not, and household income). The analyses were performed separately for each demographic grouping. The analysis based on age, for example, was performed by dividing people into three groups (see details in Section 3.1.) and calculating the appearance rate for each group; the total number of answers in each age group was divided by the sample size of each group. Those demography-based appearance rates were calculated separately for each of the 19 behavior categories and for the ‘before’ and ‘after’ conditions. The difference between the conditions were calculated by subtracting the appearance rate for the ‘before’ condition from that of the ‘after’ condition. Thus, a positive difference value indicated an increase after the pandemic onset.

One-way analysis of variance (ANOVA) was performed on the appearance rate of each behavior category to examine whether the rate differed between the subgroups of each demographic (e.g., between men and women). Since the appearance rates are discrete variables, this analysis involved converting them to arcsine square root transformation values. The details are in the end of Section 3.2.

#### 2.3.2. Participant-Based Difference Observations

The appearance-rate observations were performed based on the number of answers gathered from the whole sample. The next step of the data analysis focused on the response difference between the before and ‘after’ conditions for each participant. The number of answers in each of the 19 behavior categories were counted for each participant. Then, the values for the ‘before’ condition were subtracted from those for the ‘after’ condition to calculate the difference. The positive difference values indicated that the behaviors increased after the start of the pandemic, while the negative values indicated a decrease. The analyses described below can be replicated using the data set Supplemental S2.

Multiple regression analyses were performed on the differences between the two conditions. The analysis was performed separately for each behavior category, so the dependent variable was each participant’s difference value for the target behavior. In Section 3.3, the dependent variable was regressed onto the five demographics and the total number of answers (both conditions combined) was used as a control variable. Precisely, the predictor variables were age (continuous variable), gender (male = 1, female = 2), marital status (single = 1, married = 2), children (without = 1, with = 2) and household income (below 6 mil. = 1, above 6 mil. = 2, no answer = 3). These predictors were included in the model simultaneously. The analyses revealed which demographic influenced the behavior change.

In Section 3.4, the independent and interactive effects of age, gender, and child status were further examined. A stepwise multiple regression analysis was performed for each behavior category, for which the three prediction variables and the control variable were entered in the model at Step 1; their two-way interaction terms (age × gender, age × child, gender × child) and three-way interaction terms were included at Step 2.

In addition, similar regression analyses were performed on the magnitude of difference between the two conditions. For this analysis, the dependent variable (difference between the ‘before’ and ‘after’ conditions) was converted to absolute value to indicate the magnitude of behavior change, and the analysis was performed for each demographic. This was in order to reveal whether the magnitude of behavior change differed across subgroups of one demographic group (e.g., the overall behavior change was greater for women than for men).

Finally, Section 3.5 examined whether the magnitude of behavior change for all the behaviors combined differed depending on demographic groups. The dependent variable of this analysis was the difference in number of answers each participant reported for the before and ‘after’ condition. Total number of answers each participant produced was counted separately for the two conditions and the difference between the two was calculated. The difference was converted to absolute value to indicate the magnitude of behavior change for each participant, and this was used as the dependent variable. A multiple regression was performed on the dependent variable with the predictor variables being age, gender, marital status, having children or not, and household income. Next, separate one-way ANOVA was performed for the categorical independent variables of gender, marital status, having children or not, and household income.

### 2.4. Vaccine Attitudes in the Literature

The obtained results from the survey were contrasted with the vaccine attitudes reported in the literature. In Japan, public vaccinations started in February 2021, but were initially limited to medical workers and people above 65 years of age for a few months. It became available for all adults in June 2021 [24]. Japanese individuals’ attitudes toward the vaccine changed as time went on, but this study focused on how they were at the time of the survey, which was February 2021. Therefore, only research measuring vaccine attitudes no later than July 2021 have been referred to. According to the literature, demographic groups with hesitant attitudes were women, younger generations, individuals with low income or assets, and those without children [17,21,22,23].

## 3. Results

### 3.1. Behavior Categories and Overall Changes before and after the Pandemic Onset

A total of 1858 answers for the ‘before’ condition and 1668 answers for the ‘after’ condition were collected in the study. Three researchers grouped each answer into 19 categories, shown in Table 2. Table S1 in the Supplemental Materials summarizes the behaviors included in each category. Some answers described very unique behaviors or activities containing elements of two or more categories from Table 2; these cannot be included in any of the 19 categories and were classified as “Unable to code”.

The appearance rates of the 19 categories in the two conditions and their differences were calculated as described in Section 2.3.1, and the results are reported in Table 2. Since each participant could provide multiple answers for each category, some of the percentages reported in the table exceeded 100%. Among the categories in Table 2, those that yielded more than 10% difference (totaling nine items, marked with asterisks on the table) were carefully examined in terms of participants’ demographics: age, gender, marital status, having children or not, and household income.

Overall, some of the most noticeable behavior changes were the decrease in eating out (−64%) and the increase in prevention behaviors (+69%). Shopping was the most frequently appeared item in the ‘before’ condition (105%) and still seemed to be performed quite frequently after the virus outbreak (74%), but a significant decrease was apparent (−31%). Occasions of spending time with family and friends, traveling, and using public transport were also perceived as greatly decreased by the participants.

### 3.2. Changes by Demographics

The changes in the selected nine behavior categories were further examined by age groups (18–24, 25–65, and 66–79 years old), gender, marital status, children, and household income (below or above the annual income of 6 million yen, which roughly correspond to the average household income in Japan). Region of residence and occupation were not used because sample sizes in each sub-group of these demographics varied greatly. Target behaviors have been further reduced from the initial nine items by the following two criteria for the demographic-based examination: first, the behavior shows a greater than 10% difference between the two conditions in at least one of the groups in each demographic; second, the magnitude of the change between the two conditions noticeably differs among the sub-groups of the demographic. Table 3 summarizes the results of the behaviors meeting either of these criteria. Differences between the before and ‘after’ conditions are shown in absolute values. The table also reports the mean difference for each demographic as an absolute value.

The results by age group showed that participants in older groups reported housework more than those in the younger groups. Also, more participants mentioned this activity in the ‘after’ condition than the ‘before’ condition for all groups. Fewer participants reported leisure activities in the ‘after’ condition; this was seen in all age groups, but the magnitude of difference was noticeably large among people below 25 years of age. The reporting of shopping significantly decreased for all age groups, but the reduction was somewhat smaller for the oldest group than the other two age groups.

Regarding spending time with family and friends, the participants in the oldest group mentioned it fewer times than the other age groups in the ‘before’ condition. This is consistent with the notion that social connections shrink among elderly people [33]. The magnitude of decrease for this activity was, however, equivalent for all age groups. Mention of travel was also less frequent for the oldest group in the ‘before’ condition, maybe due to physical mobility limitations associated with aging. However, a more significant decrease was observed for the youngest group. Reporting of public transport use decreased greatly for all age groups, but the extent was more prominent for the younger groups.

The proportion of participants who reported housework, leisure activities, and spending time with family and friends are different between men and women. A higher proportion of women reported housework compared with men for the ‘before’ condition, but the overall increase was considerable for both genders. Interestingly, the reported decrease in leisure activities was only observed in women. Occasions of spending time with family and friends decreased a lot for both genders, but the responses from women indicated they performed these activities more often than men in general.

Looking at the data according to participants’ marital status, a greater number of single individuals reported behaviors relating to eating out, going shopping, and using public transport than those who were married. The extent of the decrease in those activities was also more significant for single individuals. The reporting of housework was larger for the married individuals in the ‘before’ condition, indicating that they perform housework more than singles in general. However, the difference between the ‘before’ and ‘after’ conditions was equivalent for both groups. The proportion of participants who reported spending time with family and friends decreased in the ‘after’ condition for both groups, but the magnitude was larger for the married group. Naturally, the figure in the ‘before’ condition was smaller for the single individuals, who are more likely to live alone and away from their family.

Having children impacted the behaviors of housework, shopping, and using public transport. In the ‘before’ condition, a higher proportion of participants in the no-child group reported housework and shopping than those in the with-children group. The reporting of shopping decreased in the ‘after’ condition for both groups to the equivalent extent, but the increase in housework was more salient for those without children. The number of participants who mentioned the use of public transport decreased for both groups, but the extent was much more significant for the no-child group.

Finally, household income being below or above the national average of 6 million yen influenced the reporting of housework and spending time with family and friends. More participants with lower incomes reported housework than those with higher incomes. In terms of spending time with family and friends, the greater decrease in reporting this activity was observed for the higher income group.

In addition to the activities discussed above, the pandemic outbreak massively increased prevention measures in daily life. The present results show that wearing face masks and washing hands were most practiced at the time of the survey. Interestingly, older participants did not mention prevention measures as often as younger participants. Household income also impacted the appearance rate of prevention measures, showing that 20% more participants in the higher income group reported activities in this category than those in the lower income group.

The mean difference value in Table 3 indicates the magnitude of behavior changes reported by participants in each group of the examined demographic category. In terms of age, the change appears to be greater for younger groups. While the magnitude of change does not look different depending on marital status, large differences are present for gender, having children or not, and household income. In order to confirm whether those apparent differences in mean values are present at a statistically significant level, ANOVA tests were performed on the difference values in Table 3. However, the difference values are proportion data, and there was only one datapoint for each condition to compare. These issues made it impossible to perform ANOVA on the present dependent variable, so the proportion values were transformed to alternative ones that are appropriate for the analysis. The present research used arcsine square root transformation, which has been regarded as a standard procedure to enable proportion data to be analyzed with linear methods, such as ANOVA and regression (see details in [34]). First, each of the proportion data shown in the ‘Diff.’ column in Table 3 was converted to arcsine square root transformation value (get the square-root of the proportion first, then obtain the inverse sin of that value, then subtract 0.2854 from that [35]). The mean arcsine square root value was then calculated for each demographic group. Five ANOVAs were performed comparing groups within the demographic category of age, gender, marital status, having children or not, and household income. Chi-square values were used to assess statistical significance.

For age, the magnitude of behavior changes for the young, middle, and older age groups significantly differed from each other, χ2(2) = 10.73, *p* < 0.01. Similarly, the differences between groups were significant for gender, χ2(1) = 13.30, *p* < 0.01; child status, χ2(1) = 6.67, *p* < 0.01; and income, χ2(1) = 7.97, *p* < 0.01. The within-category difference was not significant for marital status, χ2(1) = 0.27, *n.s.* The analyses showed that the younger generations, females, people without children, and high earners reported more dramatic behavioral changes than their counterparts. In the literature on vaccine attitudes, younger people, females, individuals without children, and individuals with lower incomes were reported to be relatively hesitant to vaccinate compared to their counterparts in the demographic categories.

### 3.3. Examination of the Influence of Demographics Using Multiple Regressions

The above analysis compared the proportion of participants who reported activities for each behavior category in the ‘before’ and ‘after’ conditions. The results showed that different demographics influenced different behaviors. In this section, multiple regression analyses were performed on the differences between the two conditions for each participant. The analysis was performed separately for each behavior category. The dependent variable was each participant’s difference value for the target behavior, and predictor variables were age, gender, marital status, children, and household income. Details of the analysis method is described in Section 2.3.2.

Among the five predictor variables, only age and children showed significant effects in some behavior categories. The effect of age was significant for eating out, *β* = 0.274, 95% CI [0.005, 0.018], *p* < 0.001; food delivery/takeaway, *β* = −0.174, 95% CI [−0.006, 0.000], *p* = 0.024; physical exercises, *β* = −0.190, 95% CI [−0.009, −0.001], *p* = 0.013; shopping, *β* = 0.173, 95% CI [0.001, 0.013], *p* = 0.025; staying at home, *β* = −0.173, 95% CI [−0.005, 0.000], *p* = 0.025; and using public transport, *β* = 0.169, 95% CI [0.001, 0.010], *p* = 0.025. The effect of children was significant or marginally significant for eating out, *β* = −0.143, 95% CI [−0.508, 0.027], *p* = 0.078; housework, *β* = −0.215, 95% CI [−0.584, −0.081], *p* = 0.010; physical exercises, *β* = 0.163, 95% CI [0.002, 0.330], *p* = 0.048; and watching videos, *β* = −0.208, 95% CI [−0.394, −0.050], *p* = 0.012.

Next, the difference values for each participant were converted to absolute values. The similar analyses were performed on these to investigate whether the magnitude of behavior change differed by demographics. This time, the effect of age and sex were present in some behavior categories. The effect of age was significant for eating out, *β* = −0.293, 95% CI [−0.017, −0.006], *p* < 0.001; food delivery/takeaway, *β* = −0.174, 95% CI [−0.006, 0.000], *p* = 0.024; shopping, *β* = −0.178, 95% CI [−0.011, −0.001], *p* = 0.020; staying at home, *β* = −0.173, 95% CI [−0.005, 0.000], *p* = 0.025; using public transport, *β* = −0.153, 95% CI [−0.009, 0.000], *p* = 0.045; watching videos, *β* = −0.161, 95% CI [−0.008, 0.000], *p* = 0.036; and working, *β* = −0.213, 95% CI [−0.008, −0.001], *p* = 0.006. The effect of gender was significant or marginally significant for housework, *β* = 0.127, 95% CI [−0.001, 0.354], *p* = 0.051; spending time with friends, *β* = 0.174, 95% CI [0.066, 0.401], *p* = 0.007; and watching videos, *β* = −0.139, 95% CI [−0.257, −0.012], *p* = 0.032.

### 3.4. Examination of the Influence of Interactions between Demographics Using Multiple Regressions

The analyses in Section 3.3. revealed the influential demographics on behavior changes are age, gender, and having children or not. There is a possibility that these demographics have interactive effects on the dependent variable. Thus, a stepwise multiple regression analysis was performed for each behavior category using the differences between the two conditions for each participant (not absolute values) as the dependent variable. Age, gender, and child status were used as predictors. The total number of answers for each participant was used as the control variable. In the analysis, these predictor and control variables were included in the regression model at Step 1, and then the two-way interaction terms (age × gender, age × child, gender × child) and the three-way interaction terms were entered at Step 2.

The following five behavior categories showed the independent effects of demographics without significant interactions: eating out, *Adjusted R*^2^ = 0.057, *F* (6, 314) = 4.23, *p* < 0.001; physical exercises, *Adj.R*^2^ = 0.050, *F* (6, 314) = 2.76, *p* = 0.012; shopping, *Adj.R*^2^ = 0.042, *F* (6, 314) = 2.30, *p* = 0.035; using public transport, *Adj.R*^2^ = 0.054, *F* (6, 314) = 4.02, *p* < 0.001; and watching videos, *Adj.R*^2^ = 0.028, *F* (6, 314) = 2.52, *p* = 0.021. The age and child status were the significant predictors, and the results are reported in Section 3.3.

The model with interaction terms was significant for food delivery/takeaway, *Adj.R*^2^ = 0.058, *F change* (4, 320) = 3.43, *p for R*^2^ *change* (Δ*R*^2^) = 0.009. In this model, the effect of gender, *β* = 0.260, 95% CI [0.003, 0.381], *p* = 0.047; the age × child interaction, *β* = −0.401, 95% CI [−0.020, −0.003], *p* = 0.008; and gender × child interaction, *β* = −0.473, 95% CI [−0.650, −0.154], *p* = 0.002, were significant.

Next, similar analyses performed on the absolute change values between the two conditions for each participant. The following seven behavior categories revealed the independent effects of demographics without significant interactions: eating out, *Adj.R*^2^ = 0.061, *F* (6, 314) = 4.46, *p* < 0.001; shopping, *Adj.R*^2^ = 0.032, *F* (6, 314) = 2.77, *p* = 0.012; spending time with family and friends, *Adj.R*^2^ = 0.039, *F* (6, 314) = 3.14, *p* = 0.005; using public transport, *Adj.R*^2^ = 0.037, *F* (6, 314) = 3.07, *p* = 0.006; watching videos, *Adj.R*^2^ = 0.022, *F* (6, 314) = 2.21, *p* = 0.042; and working, *Adj.R*^2^ = 0.021, *F* (6, 314) = 2.13, *p* = 0.050. The age and gender were the significant predictors, and the results are reported in Section 3.3.

The model with interaction terms was significant for food delivery, *Adj.R*^2^ = 0.058, *F change* (4, 320) = 3.43, *p for* Δ*R*^2^ = 0.009; and travel, *Adj.R*^2^ = 0.024, *F change* (4, 320) = 2.66, *p for* Δ*R*^2^ = 0.033. For the food delivery model, the effect of gender, *β* = 0.260, 95% CI [0.003, 0.381], *p* = 0.047; and of child status, *β* = 0.330, 95% CI [0.064, 0.425], *p* = 0.008, were found. In addition, the age × child interaction, *β* = −0.401, 95% CI [−0.020, −0.003], *p* = 0.008; and gender × child interaction, *β* = −0.473, 95% CI [−0.650, −0.154], *p* = 0.002, were significant. The model for travel behaviors showed a significant effect of age, *β* = −0.430, 95% CI [−0.020, −0.005], *p* = 0.001; the age × child interaction, *β* = 0.443, 95% CI [0.006, 0.032], *p* = 0.004; and the three-way interaction *β* = −0.346, 95% CI [−0.040, −0.003], *p* = 0.023.

### 3.5. Magnitude of Behavior Change by Demographics

To assess whether the magnitude of change for all behaviors differs depending on demographics, mean absolute change value for all 19 behaviors combined was calculated for each participant. Using the mean absolute change values as a dependent variable, a multiple regression analysis was performed with the five demographics as predictors. The effect of age was significant (*β* = −0.245, 95% CI [0.001, −0.004], *p* = 0.001), indicating that younger participants showed the greater magnitude of behavior change. No other effect was significant. However, the rest of the predictor variables were categorical, so the difference between the groups in each demographic was assessed using one-way ANOVA. For gender, the mean absolute change value was 0.25 (*SD* = 0.18) for men and 0.31 (*SD* = 0.18) for women, and they were significantly different from each other, *F* (1, 319) = 6.76, *p* = 0.01, $n_{p}^{2}$ = 0.021. The effect of marital status was marginally significant, *F* (1, 319) = 3.19, *p* = 0.075, $n_{p}^{2}$ = 0.01. Single participants (*Mean* = 0.31, *SD* = 0.19) showed the greater magnitude of change than those who were married (*Mean* = 0.27, *SD* = 0.19). For household income, participants who did not answer this question was omitted from the analysis. The effect was significant, *F* (1, 253) = 6.87, *p* = 0.009, $n_{p}^{2}$ = 0.026, indicating that participants with income exceeding the national average (*Mean* = 0.33, *SD* = 0.17) showed the greater magnitude of change than those with lower income (*Mean* = 0.27, *SD* = 0.19). Finally, the effect of child was not significant, *F* (1, 319) = 1.73, *p* = 0.19, $n_{p}^{2}$ = 0.005.

## 4. Discussion

The present research asked Japanese individuals to report daily behaviors they often engaged in before and after the COVID-19 outbreak to examine how behavioral changes differ by demographics. Eating out, shopping, doing housework, and engaging in leisure activities were the most frequently mentioned actions, and the pandemic seems to have significantly changed how frequently individuals perform them. Those changes are, however, mediated by demographic characteristics. The present study examined the behavior change using two types of dependent variables: the appearance rates and the conditional differences of the number of answers reported by each participant. Although these examinations showed slightly different pictures, both highlighted interesting aspects. Thus, they are discussed separately below.

### 4.1. Discussion for the Appearence Rate Observations

Examination by age showed that younger individuals generally go out more than older people; younger individuals experienced a greater reduction in these activities after the outbreak. The amount of leisure activity also reduced more greatly among younger generations. It may be because more mature people could substitute restricted leisure activities (e.g., partying, going to concerts, and joining public events) with unrestricted private activities (e.g., gardening and going for a drive). They could also substitute behaviors in crowded places with those in non-crowded places (e.g., fishing and camping). Those substituted leisure activities often require some equipment or sufficient private space in the house; these may be harder to obtain for the young population, who tend to live in small apartments and have less money. The results also revealed that the younger group had to restrict themselves more than the older group in using public transport. Car ownership would be more common among older generations; thus, they could still go out while avoiding crowds. Generally, the age-related results imply that many younger individuals had to restrict themselves from going out without finding alternative activities. Such restriction may be psychologically detrimental for them [36,37].

Housework is highlighted by all the demographic-based examinations, indicating that housework is a major part of people’s lives and its increase due to the pandemic has a significant impact. The unbalanced distribution of housework between men and women has been a serious social problem in Japan, reflecting the country’s low level of gender equality [20]. The present results showed that women reported a greater number of housework-related activities than men. At the same time, only female participants realized the decrease in leisure activities, which may relate to their extra housework, limiting their free time for leisure. These findings correspond to other studies suggesting that many women have reported pandemic-induced mental health problems [38,39,40].

Having children impacted the behaviors of housework, shopping, and using public transport. In general, participants with children had more housework and shopping than those without, but the magnitude of change for housework was greater for the latter. Notably, the use of public transport decreased more significantly for the no-children group than the with-children group. It may relate to car ownership; individuals with children might have had more access to travel without relying on public transport. Overall, the present result implies that individuals without children experienced greater behavior change (or behavioral restrictions).

In terms of household income, more participants with lower incomes reported housework than those with higher incomes. The poorer group may spend more time at home, likely for financial reasons. Limited finances may also make them refrain from outsourcing housework or childcare. A greater decrease in spending time with friends and family was observed for the higher income group, but it is because that group reported more of those activities in the ‘before’ condition than the lower income group. The appearance rate of this activity was similar for both groups in the ‘after’ condition. This may be because the lower income group contained more young and single individuals, who are more likely to be living independently.

Interestingly, activities related to internet use and sedentary behaviors (e.g., watching TV), which have been reported to be increased by the pandemic [7], were not mentioned very often in the present study. No significant change between the two conditions was also observed. Although the present results do not strictly reflect the actual frequency of activity performance, it can be said that the participants were not conscious of the changes in those activities. It suggests that the impact of increased internet use on mental health might not be as significant as other causes, at least in Japan, despite an established direct relationship between the two factors.

In terms of the magnitude of behavior changes, the appearance-rate observations conclude that the greatest change was experienced by women, younger generations, those without children, and those with higher incomes compared with the other demographic groups.

### 4.2. Discussion for the Participant-Based Difference Observations

The outcomes of the participant-based examinations must be interpreted cautiously in the present research. The most important reason for that is the response bias. In the study, all participants firstly wrote activities for the ‘before’ condition. Possibly due to fatigue or loss of attention, the total number of answers for the ‘after’ condition was smaller than the ‘before’ condition. This bias was corrected in the appearance-rate observations, because the rate reported in Table 2 and Table 3 were calculated for each condition. On the contrary, this bias could not be fully considered for the regression analyses, which used the difference between the two conditions for each participant, although total number of answers was used as a control variable. In addition, because of the order of the conditions, some participants showed a strong tendency to copy what they wrote for the ‘before’ condition to the ‘after’ condition. Therefore, it is likely that the answers from those participants did not reflect their behavior changes accurately. All in all, the data of the participant-based observations must have been more severely influenced by the response biases and the methodological constraints compared with the appearance-rate observations.

Nevertheless, the demographics of age and child status significantly influenced the number of activities reported in the study. Age, eating out, shopping, and using public transport had a positive beta value, meaning that the older participants reported more changes in a positive direction. Importantly, it is unlikely that the older participants started to perform these activities more after the pandemic onset because the overall number of these answers decreased, as shown in Table 2. Therefore, it is more appropriate to interpret that the greater reduction of these behaviors is observed for the younger participants. The activities of physical exercises and staying at home showed negative beta values. This can be interpreted as the younger individuals’ greater increase of these activities or the older individuals’ greater decrease of them. An interactive effect of age and child status was found for food delivery, and this indicates that the younger participants with children showed a greater increase in this activity. Younger parents are presumed to have younger children, who cannot prepare their own food. Therefore, the extent of reliance in food delivery is not simply influenced by the child status but also by parents’ age (or children’s age).

Comparing the appearance-rate observation and the participant-based observation, some of the activities showing significant differences did not match. However, the overall trend of the age-related behavior change is consistent. The activities involving movement and human contact were more greatly restricted in the younger participants, represented by the decrease of public transport use and eating out. Furthermore, they reported increase in activities related to illness prevention, such as social distancing (staying at home), after the pandemic onset. This corresponds to the large decrease in leisure activities and traveling, and the large increase in prevention behaviors observed among the younger participants in the appearance-rate observation.

In terms of the magnitude of behavior change, the results statistically confirmed that it was greater among younger participants. For the absolute change value, the age influenced traveling behaviors in such a way that the younger participants showed the greater change. However, the age and child status interactively affected this behavior. Among the younger individuals, the change was greater for those without children. This may be because traveling without children is easier, and those participants traveled more than those with children before the pandemic. Interestingly, the two-way interaction was further influenced by gender, indicating that behavior change with regard to traveling was under a complex influence of demographics.

The influence of having children was observed for eating out, housework, physical exercises, and watching videos. Apart from physical exercises, people without children increased those behaviors after the pandemic onset. The interpretation of eating out again requires caution. Rather than interpreting that individuals without kids started to eat out more, it is more likely that those with children greatly reduced this activity. As in Table 3, housework was also picked up by the appearance-rate observation. The decrease in public transport use among the child-free group was found in the appearance-rate observation as well. Overall, the results indicate that participants in the no-child group have increased the behaviors performed alone, avoiding human contact. This may result in the increased time for watching videos at home and housework.

The child status interacted with age and gender to influence behaviors of food delivery and traveling. Food delivery generally increased after the pandemic onset, but the magnitude appears to be more significant for individuals with children. However, among those with children, younger people and men showed the greater change. The effect on younger parents was already discussed above, and the impact on men may be explained by distribution of housework between men and women. Women were likely to have experienced an increase in housework after the pandemic onset (see below). Therefore, many men might have taken over the role of food preparation in the family to help women. Despite these group differences, the magnitude of change across all the behaviors was not affected by the presence of children.

The effect of gender was shown for the activity-based analyses when the absolute change values were used as the dependent variable. It appears that the change in housework and spending time with friends was greater for women than men, while the change in watching videos was greater for men. As a general trend, housework and watching videos increased after the start of the pandemic, and spending time with family and friends decreased. Therefore, women must have experienced greater increase in housework than men, while experiencing a greater decrease in spending time with family and friends. At the same time, men must have experienced a greater increase in watching videos compared with women. The univariate ANOVA in Section 3.5 showed that the magnitude of behavior change across all the behaviors was more significant for women than men.

Finally, the effect of household income was not found for any of the activities, but the overall magnitude of behavior change was influenced by this demographic. The participants with higher incomes showed the greater magnitude of behavior change than those with lower incomes.

Although the detailed results differ between the appearance rate and participant-based difference observations, it is fair to conclude that the participant-based examinations generally support the account that the greater change was experienced by women, younger generations, those without children, and those with higher incomes than their demographic counterparts.

### 4.3. Behavior Change and Vaccine Attitudes

Participants who reported the greater magnitude of behavior change in the present study were women, younger generations, those without children, and those with higher incomes. Apart from income, those demographic categories correspond to the population who reported a hesitant attitude towards the vaccines at the early stage of the Japanese vaccine program [17,21,22,23]. It can be interpreted that individuals who were unwilling to receive vaccines were more motivated to prevent virus transmission through behavior change. In contrast, those who were reluctant to change behaviors were more willing to receive vaccines. It is reported that the most common reasons for vaccine hesitancy in Japan were side effects and uncertain safety [23]. Individuals accepting these risks appeared to have prioritized maintaining pre-pandemic behaviors as much as possible, while those who were unwilling to take risks associated with the vaccines chose to prevent the illness through behavior changes.

In the present study, participants with higher household incomes reported a greater magnitude of behavior change (albeit only for three behaviors) than those with lower incomes. However, some literature reports that the latter group is more hesitant toward vaccines [16,17]. Crucially, the findings on household income have been inconsistent, at least in Japan. While Machida et al. [17] found more vaccine hesitance among the lower income population, Kadoya et al. [21] found no relationship between household income and vaccine hesitancy. According to Kadoya et al. [21], household asset value is more influential for vaccine attitudes than income. A large Japanese survey that investigated vaccine recipients in September 2021 also reported that household income did not impact vaccine reception, while vaccination rates for younger individuals, women, and singles were lower than those for older people, men, and married individuals [24]. In the present study, the impact of household income was much less robust than the effect of age, gender, and child status. Therefore, the impact of household income on vaccine attitude and its relationship with behavior changes are inconclusive.

### 4.4. Limitations and Future Research

Overall, the behavior changes measured with a free-response survey revealed a dynamic change in people’s daily behavior and how demographic characteristics mediated this. Also, the behavior change appears to be related to vaccine attitudes in a logically explainable way. However, this study contains a number of limitations. First, participants’ answers did not directly reflect how often those behaviors were performed. The more conventional studies on behavior change tend to ask participants to report how often they perform certain behavior using a Likert scale [4,5,6,19,20]. This is the optimum procedure to measure the frequency of the behavior precisely, and this enables researchers to compare the ‘before’ and ‘after’ condition for the same behavior directly. In addition, the dependent variable of the present research, which was the number of answers each participant produced, had only limited variations between the conditions. The maximum number of answers participants could produce was restricted to ten for each condition. The majority of participants stopped answering once they had written five, which was the minimum requirement. With this procedure, the deviation range of the dependent variable was strictly limited, especially compared with the variable taken with the Likert scale. Therefore, any quantitative analyses performed on the present data will struggle to reach a statistically significant level. This is the reason why the present research reported the appearance-rate results. In order to resolve this issue, the free-response method can be altered slightly. For example, responses can be gathered separately for pre-determined behavior categories, so that all participants have responses for all the categories used for the statistical analyses (in the present study, each participant reported for only a few categories out of the 19 that were used for the analysis). Eliminating limits or requirements for the number of answers may also increase the variability of the dependent variable.

Along with those limitations, the free-response measure had some positive points. The present research identified 19 behavior categories without exhausting participants, while answering the Likert-style questions 19 times is tiresome. Furthermore, the free-response answer must be a collection of activities that participants were clearly conscious about performing. For example, the present result did not find any change for the usage of internet and electronic devices; however, many other studies showed there was an increase for those activities after the pandemic onset [7]. The increase could well be the case for the present participants, but it was not shown in their answers because they were not very aware of those behaviors. In contrast, activities such as shopping and housework appeared to be important for most participants, and it can be easily interpreted that the changes that occurred for those activities had significant impacts on their daily lives. Such subjective importance cannot be measured if the study asked participants to rate how often they perform a pre-selected activity.

In terms of the assessment of vaccine attitudes, the present research has a few shortcomings. The vaccine attitudes for particular demographics were only taken from the literature, not measured from the present sample. Participants’ knowledge about vaccines was also unknown. In addition, other personal details, such as health conditions, education level, and the size of an individual’s social network were not recorded or considered in the present study. Those factors not only impact vaccine attitudes, but also significantly affect the trajectory of epidemic progress [27]. In other words, they are key factors for an effective vaccination strategy [29,30]. Although the health, education, and socialization situations for individuals can be estimated from their demographics to some extent (e.g., younger people tend to be healthier and have larger social networks), they should still vary within each demographic category. Therefore, including those measurements is essential for future research. Due to the absence of those variables in the present study, the suggested theory about the relationship between the behavior change and vaccine attitudes may better be regarded as exploratory.

## 5. Conclusions

The present research investigated behavioral changes brought about by the COVID-19 pandemic among Japanese citizens using a free-response survey in which participants described their frequently performed behaviors. The results revealed that the pandemic altered various daily behaviors, but an individual’s demographics, which in part determines their lifestyle, modulated the impact. The younger generations, women, and people without children showed a significantly greater magnitude of behavior change than their counterparts in each demographic category. They were the groups with more hesitant attitudes toward vaccines [17,21,22,23]. The COVID-19-related behavior changes and vaccine attitudes are highly likely to be related in such a way that people who are reluctant to change behaviors are willing to receive vaccines, while those who are concerned about vaccination risks favor behavior changes for illness prevention.

The vaccine program in Japan has been successful, and vaccine hesitance is not a social problem in the country nowadays. Tokiya and colleagues report the gradual increase of vaccine acceptance attitudes among Japanese since the start of the program [41]. Hesitant vaccine attitudes were in the minority at the time of booster administration (started from November 2021), and one of the most important reasons for the increased acceptance is that receiving vaccines has become a social norm [42]. Therefore, the findings of the present research may not directly contribute to the vaccine strategy for the current epidemic situation. However, retrospectively studying pandemic-related psychology and behaviors provides significant references and guidelines for future events that occur under similar circumstances.

## Figures and Tables

**Table 1:** Summary of the dependent variables and statistical tests reported in the result section.

Section	Dependent Variable	Independent Variable	Statistical Test	Purpose
Section 3.1.	Appearance rates (AR)	The conditions (before and after)	None	Assessing the difference between the conditions
Section 3.2.	AR by the demographics ^1^	The conditions	None	Assessing the difference between the conditions by demographics
Section 3.2.	Difference (Diff) in AR in absolute value (AV)	Subgroups of each demographic	One-way ANOVA for each dem	Examining whether the magnitude of behavior change differ between subgroups of each demographic
Section 3.3.	Diff in number of answers between the conditions calculated per participant (Part), per behavior category (BC)	Demographics	Multiple regression for each BC	Examining how demographics influenced the behavior change
Section 3.3	Diff in answers between the conditions in AV, calculated per Part, per BC	Demographics	Multiple regression for each BC	Examining whether the magnitude of behavior change differed by demographics
Section 3.4	Diff in answers between the conditions calculated per Part, per BC.	Demographics (age, gender, child status)	Stepwise multiple regression for each BC	Examining the interactive effects of demographics on behavior change
Section 3.4	Diff in answers between the conditions in AV, calculated per Part, per BC	Demographics (age, gender, child status)	Stepwise multiple regression for each BC	Examining the interactive effects of demographics on the magnitude of behavior change
Section 3.5	Diff in answers between the conditions in AV, calculated per Part, all behaviors	Demographics	Multiple regression	Examining whether the magnitude of behavior change is influenced by the demographics
Section 3.5	Diff in number of answers between the conditions in AV, calculated per Part, all behaviors	Subgroups of each demographic	One-way ANOVA for each demographic	Examining whether the magnitude of behavior change differed between subgroups of each demographic

^1^ The demographics are age, gender, marital status, having children or not, and household income.

**Table 2:** Appearance rates for the 19 behavior categories in the ‘before’ and ‘after’ conditions.

Category	Before ^1^	After	Change ^2^
Eat at home	8%	16%	8%
Eating out *^3^	92%	29%	−63%
Food delivery, take away	0%	9%	9%
Going out *	19%	6%	−13%
Housework *	60%	76%	16%
Leisure activities *	55%	41%	−14%
Life necessities	24%	27%	3%
Pandemic prevention *	4%	71%	67%
PCs, smartphones, internet	9%	14%	5%
Physical exercises	29%	31%	2%
Reading, listening to music	6%	11%	5%
Shopping *	102%	73%	−30%
Spending time with family and friends *	37%	12%	−25%
Staying at home	0%	9%	9%
Studying	5%	1%	−4%
Travel *	35%	10%	−25%
Using public transport *	43%	16%	−27%
Watching videos	22%	30%	8%
Working, schooling	13%	20%	7%
Unable to code	5%	5%	0%

^1^ The appearance rate for each condition was calculated as follows: the total number of answers in each behavior category/number of participants (*N* = 321) ^2^ The change column shows the difference in appearance rate between the conditions (after–before) ^3^ The items with asterisks were further examined by demography.

**Table 3:** Appearance rates for the selected behaviors by demographic.

Age	18–24 (*N* = 37)			25–65 (*N* = 191)			66–79 (*N* = 93)		
	Bef.^1,2^	Aft.^1^	Diff.^1,3^	Bef.	Aft.	Diff.	Bef.	Aft.	Diff.
Housework	24%	38%	14%	59%	77%	18%	80%	92%	12%
Leisure activities	76%	35%	41%	49%	37%	12%	61%	56%	5%
Pandemic prevention	0%	97%	97%	5%	81%	76%	4%	45%	40%
Shopping	73%	38%	35%	114%	79%	35%	97%	78%	19%
Spending time with family/friends	38%	22%	16%	42%	10%	32%	29%	12%	17%
Travel	41%	5%	36%	38%	12%	26%	29%	6%	23%
Using public transports	57%	5%	52%	49%	19%	30%	29%	16%	13%
Mean difference (absolute value)			41.57%			32.71%			18.42%
Gender	Male (*N* = 160)			Female (*N* = 161)					
	Bef.	Aft.	Diff.	Bef.	Aft.	Diff.			
Housework	39%	54%	15%	83%	101%	18%			
Leisure activities	56%	53%	3%	55%	31%	24%			
Pandemic prevention	4%	63%	59%	4%	83%	79%			
Spending time with family/friends	23%	7%	16%	52%	17%	35%			
Mean difference (absolute value)			23.25%			39%			
Marital status	Single (*N* = 128)			Married (*N* = 193)					
	Bef.	Aft.	Diff.	Bef.	Aft.	Diff.			
Eating out	117%	42%	75%	78%	22%	56%			
Housework	49%	68%	19%	69%	84%	15%			
Leisure activities	70%	66%	4%	46%	26%	20%			
Pandemic prevention	5%	78%	73%	4%	69%	65%			
Shopping	136%	94%	42%	84%	61%	23%			
Spending time with family/friends	28%	9%	19%	44%	15%	29%			
Using public transports	54%	24%	30%	37%	11%	26%			
Mean difference (absolute value)			37.43%			33.43%			
Children	Without (*N* = 149)			With (*N* = 172)					
	Bef.	Aft.	Diff.	Bef.	Aft.	Diff.			
Housework	43%	72%	29%	76%	82%	6%			
Pandemic prevention	5%	74%	69%	3%	71%	68%			
Shopping	91%	60%	31%	117%	86%	31%			
Using public transports	51%	13%	38%	38%	20%	18%			
Mean difference (absolute value)			41.75%			30.75%			
Household income	Below 6 M (*N* = 169)			Above 6 M (*N* = 86)					
	Bef.	Aft.	Diff.	Bef.	Aft.	Diff.			
Housework	69%	86%	17%	40%	59%	19%			
Pandemic prevention	2%	62%	60%	0%	84%	84%			
Spending time with family/friends	31%	12%	19%	50%	9%	41%			
Mean difference (absolute value)			32%			48%			

^1^ Bef., Aft. and Diff.: before, after, and difference, respectively. ^2^ The appearance rate for each condition was calculated as follows: the total number of answers for each subgroup of demographic in each behavior category/number of participants in each demographic subgroup (indicated by *N*) ^3^ The Diff. column shows the differences in appearance rates between the conditions (after–before) converted to absolute values.

## Data Availability

The data supporting reported results can be found in the Supplemental Materials at https://osf.io/t4hau/ (accessed on 15 December 2022).

## Supplementary Materials

The following supporting information can be downloaded at: https://www.mdpi.com/article/10.3390/vaccines11010192/s1, An excel file, Supplemental S1 and a SPSS data file, Supplemental S2, are available online at https://osf.io/t4hau/ (accessed on 15 December 2022). Supplemental S1 consists of a raw dataset and Table S1: Number of responses and appearance rates of the behaviors classified in to 19 categories. The SPSS data file is the one authors used to perform regression analyses (see Section 2.3.2).

## References

[1] Nelson B.W., Pettitt A., Flannery J.E., Allen N.B. (2020). Rapid assessment of psychological and epidemiological correlates of COVID-19 concern, financial strain, and health-related behavior change in a large online sample. PLoS ONE.

[2] Sjödin H., Wilder-Smith A., Osman S., Farooq Z., Rocklöv J. (2020). Only strict quarantine measures can curb the coronavirus disease (COVID-19) outbreak in Italy, 2020. Eurosurveillance.

[3] West R., Michie S., Rubin G.J., Amlôt R. (2020). Applying principles of behaviour change to reduce SARS-CoV-2 transmission. Nat. Hum. Behav..

[4] Chang Y.-K., Hung C.-L., Timme S., Nosrat S., Chu C.-H. (2020). Exercise behavior and mood during the COVID-19 pandemic in Taiwan: Lessons for the future. Int. J. Environ. Res. Public Health.

[5] Meyer J., McDowell C., Lansing J., Brower C., Smith L., Tully M., Herring M. (2020). Changes in physical activity and sedentary behavior in response to COVID-19 and their associations with mental health in 3052 US adults. Int. J. Environ. Res. Public Health.

[6] Sonza A., da Cunha de Sá-Caputo D., Sartorio A., Tamini S., Seixas A., Sanudo B., Süßenbach J., Provenza M.M., Xavier V.L., Taiar R. (2021). COVID-19 lockdown and the behavior change on physical exercise, pain and psychological well-being: An international multicentric study. Int. J. Environ. Res. Public Health.

[7] Elhadi M., Alsoufi A., Msherghi A., Alshareea E., Ashini A., Nagib T., Abuzid N., Abodabos S., Alrifai H., Gresea E. (2021). Psychological health, sleep quality, behavior, and internet use among people during the COVID-19 pandemic: A cross-sectional study. Front. Psychiatry.

[8] Liu X., Luo W.-T., Li Y., Li C.-N., Hong Z.-S., Chen H.-L., Xiao F., Xia J.-Y. (2020). Psychological status and behavior changes of the public during the COVID-19 epidemic in China. Infect. Dis. Poverty.

[9] Shamsalinia A., Mohammadi S., Ghaffari F., Arazi T. (2020). Changes in preventive behavior during the first 3 months of the COVID-19 outbreak in Iran. Disaster Med. Public Health Prep..

[10] Wang C., Pan R., Wan X., Tan Y., Xu L., Ho C.S., Ho R.C. (2020). Immediate psychological responses and associated factors during the initial stage of the 2019 coronavirus disease (COVID-19) epidemic among the general population in China. Int. J. Environ. Res. Public Health.

[11] Barber S.J., Kim H. (2021). COVID-19 worries and behavior changes in older and younger men and women. J. Gerontol. B.

[12] Lee M., You M. (2020). Psychological and behavioral responses in South Korea during the early stages of coronavirus disease 2019 (COVID-19). Int. J. Environ. Res. Public Health.

[13] Dinić B.M., Bodroža B. (2021). COVID-19 protective behaviors are forms of prosocial and unselfish behaviors. Front. Psychol..

[14] Bauman A.E., Reis R.S., Sallis J.F., Wells J.C., Loos R.J., Martin B.W. (2012). Correlates of physical activity: Why are some people physically active and others not?. Lancet.

[15] Our World in Data Coronavirus (COVID-19) Vaccinations. https://ourworldindata.org/covid-vaccinations?country=JPN.

[16] Nomura S., Eguchi A., Yoneoka D., Murakami M., Ghaznavi C., Gilmour S., Kaneko S., Kawashima T., Kunishima H., Naito W. (2022). Characterising reasons for reversals of COVID-19 vaccination hesitancy among Japanese people: One-year follow-up survey. Lancet Reg. Health West. Pac..

[17] Machida M., Nakamura I., Kojima T., Saito R., Nakaya T., Hanibuchi T., Takamiya T., Odagiri Y., Fukushima N., Kikuchi H. (2021). Acceptance of a COVID-19 Vaccine in Japan during the COVID-19 Pandemic. Vaccines.

[18] Latkin C.A., Dayton L., Yi G., Colon B., Kong X. (2021). Mask usage, social distancing, racial, and gender correlates of COVID-19 vaccine intentions among adults in the US. PLoS ONE.

[19] Azizi A., Achak D., Aboudi K., Saad E., Nejjari C., Nouira Y., Hilali A., Youlyouz-Marfak I., Marfak A. (2020). Health-related quality of life and behavior-related lifestyle changes due to the COVID-19 home confinement: Dataset from a Moroccan sample. Data Brief.

[20] Rossinot H., Fantin R., Venne J. (2020). Behavioral changes during COVID-19 confinement in France: A web-based study. Int. J. Environ. Res. Public Health.

[21] Kadoya Y., Watanapongvanich S., Yuktadatta P., Putthinun P., Lartey S.T., Khan M.S.R. (2021). Willing or hesitant? A socioeconomic study on the potential acceptance of COVID-19 vaccine in Japan. Int. J. Environ. Res. Public Health.

[22] Khan M.S.R., Watanapongvanich S., Kadoya Y. (2021). COVID-19 vaccine hesitancy among the younger generation in Japan. Int. J. Environ. Res. Public Health.

[23] Okamoto S., Kamimura K., Komamura K. (2022). COVID-19 vaccine hesitancy and vaccine passports: A cross-sectional conjoint experiment in Japan. BMJ Open.

[24] Okubo T., Inoue A., Sekijima K. (2021). Who got vaccinated for COVID-19? Evidence from Japan. Vaccines.

[25] Yoneoka D., Eguchi A., Nomura S., Kawashima T., Tanoue Y., Murakami M., Sakamoto H., Maruyama-Sakurai K., Gilmour S., Shi S. (2022). Identification of optimum combinations of media channels for approaching COVID-19 vaccine unsure and unwilling groups in Japan. Lancet Reg. Health West. Pac..

[26] Arenas A., Cota W., Gómez-Gardeñes J., Gómez S., Granell C., Matamalas J.T., Soriano-Paños D., Steinegger B. (2020). Modeling the spatiotemporal epidemic spreading of COVID-19 and the impact of mobility and social distancing interventions. Phys. Rev. X.

[27] Britton T., Ball F., Trapman P. (2020). A mathematical model reveals the influence of population heterogeneity on herd immunity to SARS-CoV-2. Science.

[28] Devaraj J., Madurai Elavarasan R., Pugazhendhi R., Shafiullah G.M., Ganesan S., Jeysree A.K., Khan I.A., Hossain E. (2021). Forecasting of COVID-19 cases using deep learning models: Is it reliable and practically significant?. Results Phys..

[29] Krueger T., Gogolewski K., Bodych M., Gambin A., Giordano G., Cuschieri S., Czypionka T., Perc M., Petelos E., Rosińska M. (2022). Risk assessment of COVID-19 epidemic resurgence in relation to SARS-CoV-2 variants and vaccination passes. Commun. Med..

[30] Markovič R., Šterk M., Marhl M., Perc M., Gosak M. (2021). Socio-demographic and health factors drive the epidemic progression and should guide vaccination strategies for best COVID-19 containment. Results Phys..

[31] Arthur R.F., Jones J.H., Bonds M.H., Ram Y., Feldman M.W. (2021). Adaptive social contact rates induce complex dynamics during epidemics. PLoS Comput. Biol..

[32] Perra N. (2021). Non-pharmaceutical interventions during the COVID-19 pandemic: A review. Phys. Rep..

[33] Antonucci T.C., Ajrouch K.J., Birditt K.S. (2014). The convoy model: Explaining social relations from a multidisciplinary perspective. Gerontologist.

[34] Warton D.I., Hui F.K.C. (2011). The arcsine is asinine: The analysis of proportions in ecology. Ecology.

[35] Mrdkoff T. Standard Transformations—Psychological and Brain Sciences. http://www2.psychology.uiowa.edu.

[36] Glowacz F., Schmits E. (2020). Psychological distress during the COVID-19 lockdown: The young adults most at risk. Psychiatry Res..

[37] Ueda M., Stickley A., Sueki H., Matsubayashi T. (2020). Mental health status of the general population in Japan during the COVID-19 pandemic. Psychiatry Clin. Neurosci..

[38] Proto E., Quintana-Domeque C. (2021). COVID-19 and mental health deterioration by ethnicity and gender in the UK. PLoS ONE.

[39] Ruppanner L., Tan X., Carson A., Ratcliff S. (2021). Emotional and financial health during COVID-19: The role of housework, employment and childcare in Australia and the United States. Gend. Work. Organ..

[40] Stroud I., Gutman L.M. (2021). Longitudinal changes in the mental health of UK young male and female adults during the COVID-19 pandemic. Psychiatry Res..

[41] Tokiya M., Hara M., Matsumoto A., Ashenagar M.S., Nakano T., Hirota Y. (2022). Association of vaccine confidence and hesitancy in three phases of COVID-19 vaccine approval and introduction in Japan. Vaccines.

[42] Tokiya M., Hara M., Matsumoto A., Ashenagar M.S., Nakano T., Hirota Y. (2022). Acceptance of booster COVID-19 vaccine and its association with components of vaccination readiness in the general population: A cross-sectional survey for starting booster dose in Japan. Vaccines.

